# Investigation of potential artefactual changes in measurements of impedance changes during evoked activity: implications to electrical impedance tomography of brain function

**DOI:** 10.1088/0967-3334/36/6/1245

**Published:** 2015-05-26

**Authors:** Kirill Y Aristovich, Gustavo S Dos Santos, David S Holder

**Affiliations:** Department of Medical Physics and Biomedical Engineering, University College London, Malet Place Engineering Building, Gower Street, London, WC1E 6BT, UK; k.aristovich@ucl.ac.uk

**Keywords:** bioimpedance, evoked activity, EIT, nerve, compound action potential

## Abstract

Electrical impedance tomography (EIT) could provide images of fast neural activity in the adult human brain with a resolution of 1 ms and 1 mm by imaging impedance changes which occur as ion channels open during neuronal depolarization. The largest changes occur at dc and decrease rapidly over 100 Hz. Evoked potentials occur in this bandwidth and may cause artefactual apparent impedance changes if altered by the impedance measuring current. These were characterized during the compound action potential in the walking leg nerves of *Cancer pagurus*, placed on Ag/AgCl hook electrodes, to identify how to avoid artefactual changes during brain EIT. Artefact-free impedance changes (*δZ*) decreased with frequency from −0.045 ± 0.01% at 225 Hz to −0.02 ± 0.01% at 1025 Hz (mean ± 1 SD, *n* = 24 in 12 nerves) which matched changes predicted by a finite element model. Artefactual *δZ* reached c.300% and 50% of the genuine membrane impedance change at 225 Hz and 600 Hz respectively but decreased with frequency of the applied current and was negligible above 1 kHz. The proportional amplitude (*δZ* (%)) of the artefact did not vary significantly with the amplitude of injected current of 5–20 *µ*A pp. but decreased significantly from −0.09 ± 0.024 to −0.03 ± 0.023% with phase of 0 to 45°. For fast neural EIT of evoked activity in the brain, artefacts may arise with applied current of >10 *µ*A. Independence of *δZ* with respect to phase but not the amplitude of applied current controls for them; they can be minimized by randomizing the phase of the applied measuring current and excluded by recording at >1 kHz.

## 1. Introduction

### 1.1. Background

Electrical impedance tomography (EIT) is a medical imaging method which has the potential to yield images of neuronal depolarization in the brain, by imaging changes in impedance which occur as ion channels open (Holder 1987, Gilad *et al* 2009). In EIT, a set of electrodes is applied to the surface of the excitable tissue (brain or nerve). Typically, alternating constant amplitude current is injected through a pair of electrodes, and voltages are measured on the remaining electrodes. The technique is applied in time difference mode i.e. voltages are measured differentially with respect to the baseline, or initial time point, which results in voltage changes with respect to time. Images of the internal complex conductivity are reconstructed to yield tomographic images of the activity. Images are produced during repeated evoked activity, usually physiologically evoked responses in response to somatosensory, auditory or visual stimuli. Averaging is usually employed to increase the effective signal-to-noise ratio (SNR).

The principle behind the impedance change during neuronal activity is that current applied to excitable nervous tissue is restricted to the extracellular space at rest but then travels into the intracellular compartment as ion channels open during activity; this appears as a resistance decrease of about 1% in nerve during the compound action potential (CAP) or the brain during evoked activity (Liston *et al* 2012). Modelling indicates that this change is largest at dc and then decreases rapidly with applied current above 100 Hz. This is because applied alternating current crosses membrane capacitance at rest so that the effect of the resistance change due to ion channel opening is diluted, and this effect increases with the applied frequency, starting at about 100 Hz.

#### 1.1.1. Previous studies of impedance changes during activity in brain or nerve.

The first experiments with electrical impedance changes during neural activity were conducted in squid giant axon (Cole and Curtis 1939); significant membrane impedance decreases of up to 10% were measured. These changes were subsequently confirmed during activity in cat cortex (Freygang and Landau 1955, Galambos and Velluti 1968), spinal motoneurons (Smith *et al* 1967), and red nucleus neurons (Tsukahara and Fuller 1969).

Later, studies in crab walking leg nerves showed reproducible impedance decreases of 0.2–0.7% at dc, 0.005% at 50 kHz (Holder 1992), and 0.1–1% occurring at low frequencies of up to 600 Hz (Gilad *et al* 2009, Oh *et al* 2011). These were followed by recordings made in rat brain using a planar electrode array, placed directly over the motor cortex. Impedance decreases of 0.01–0.1% during evoked response were recorded during somatosensory evoked responses at 125–825 Hz (Oh *et al* 2011). This demonstrated the potential of EIT to produce tomographic images of evoked fast neural activity using repetitive stimuli and non-penetrating electrode grids placed on the brain surface. Human studies with scalp electrodes with an applied square wave at 1 Hz also showed significant impedance decreases of 0.001% during evoked visual and somatosensory activity. However, the signals were on the border of statistical significance. This was because of degradation of the SNR by the layers of the scalp and the skull, and necessitated long averaging times due to obscuration of the changes by the uncorrelated electroencephalography (EEG) (Gilad and Holder 2009).

In making impedance recordings in excitable tissue, the ideal is that the injected current does not excite the tissue. However, the SNR for EIT in the brain is often limited, and it is desirable to increase the applied measuring current to increase the signal. The risk in doing so is that the measuring current itself opens ion channels and produces evoked activity or else alters excitability in a sub-threshold manner. The current density levels, at which evoked activity starts to occur is > 200 A m^−2^ (Merton and Morton 1980, Liebetanz *et al* 2009, Edwards *et al* 2013), and it is unlikely that EIT would operate on such high current levels, because it would require significantly larger than 1 mA (safety limit for EIT) to be applied on the scalp, or >100 *µ*A in case of the subdural electrodes. However, the current levels used in EIT could cause the recording artefact by altering the properties of the CAP in nerve or evoked potential in the brain. The relationship between applied current and tissue excitation has been reviewed in (Gilad *et al* 2007, Reato *et al* 2013). It was concluded that significant effects on excitable tissues would occur with current densities of >0.6–1.2 A m^−2^ at frequencies up to about 1 kHz. For an electrode with a surface area of 0.5 mm^2^, which is approximately the size of silver hook electrodes used to record from nerves in the studies above, or subdural electrodes used for EIT of the brain 0.6 mm in diameter (Oh *et al* 2011), this equates to an injected impedance measuring current of about 1 *µ*A. Larger applied currents of about 10 *µ*A were used in previous studies (Gilad *et al* 2009, Oh *et al* 2011) and so might be expected to produce an artefact.

#### 1.1.2. Conventional artefacts, artefact removal technique, and possible artefacts which cannot be removed.

As mentioned above, the largest voltage response to the applied current occurs at frequencies less than 1 kHz. However the recorded voltages include changes arising from both the impedance change but also the evoked activity. The latter needs to be removed by subtraction of the separately recorded evoked signal, or controlling the phase of the injected current with respect to the stimulation, such that CAP component, which is additive and phase-locked to the stimulation, cancels during averaging (Gilad and Holder 2009). In the published studies described above, controls were undertaken to ensure the recorded changes were not artefactual. This was achieved by showing that observed changes corresponded to biophysical modelling and that the actual voltage change was proportional to applied current. If changes were impedance-related, then voltage changes should scale proportionately to the applied current. Any artefactual changes might be expected to be related non-linearly to applied current and so yield differing apparent impedance changes for different applied currents. An additional control was to verify that the evoked potentials did not alter significantly with different applied impedance measuring currents. However, if the current applied for impedance measurements alters the evoked activity, then these effects are not removed through subtraction or averaging as they present with the same frequency and phase as the applied current. Therefore these voltage changes cannot be separated from those arising from the actual impedance change. These artefactual impedance changes are the topic of this study. We refer to the recorded voltage changes due to the impedance change itself as ‘impedance-related’ and due to the confounding evoked potentials as ‘artefactual’.

**Figure 1.**
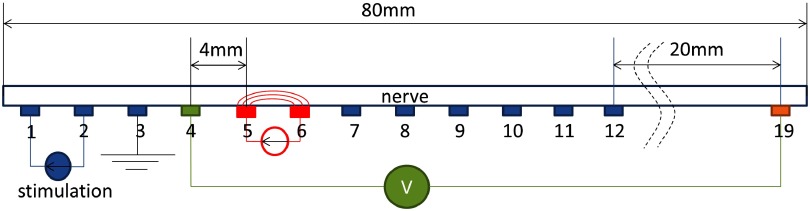
The nerve was placed on the electrode array 1:19 with the electrodes spaced 4 mm apart. Stimulation electrodes 1–2 triggered the CAP; electrode 3, the earth, removed the stimulation artefact; electrodes 4:19 were current injection or voltage measuring electrodes. In the figure, the artefact-free method is shown, with current injected between electrodes 5–6, and measurement between electrodes 4–19.

In previous recordings made in peripheral nerve, and also in present work, the artefact was avoided by the following method (figure 1) (Oh *et al* 2011). The nerve was placed on silver hooks spaced approximately 4 mm apart. The CAP was initiated by electrical stimulation at one end (electrodes 1 and 2), and the nerve was earthed at the adjacent electrode 3. The impedance measuring current was then applied distally, usually at electrodes 5 and 6. If voltage recording were to take place distally to this, then there was the risk of recording an artefact if the measuring current altered the shape or latency of the CAP. To avoid this, voltage was recorded with the first electrode placed at position 4, 4 mm proximal to the impedance measuring electrodes. As long as it was more than two space constants (the spatial distance of the action potential) proximal to the current injecting electrode 5, the recorded CAP was not significantly affected by any current injected at electrode 5. The other voltage recording electrode still in theory would have recorded any altered action potential and so would have yielded an artefact, as it was distal to the impedance current injecting electrode pair. This was avoided by recording with a distant electrode—position 19 in this example (Olney *et al* 1987). These controls were put in place (see experimental design section) in order to make sure that the artefact-free recordings were valid.

Unfortunately, this approach cannot be used directly for recording impedance changes during evoked activity in the brain, as it is not possible to place one recording electrode proximal to the impedance current injecting electrodes. However, studying the artefactual impedance in response to various parameters, such as phase, amplitude, and frequency of the applied current in a simple model allows to identify possible other ways for avoiding the artefact.

### 1.2. Purpose

Although the eventual intended application of EIT is in the brain, recording in peripheral nerve provides a convenient model for determining proof of principle, validating biophysical modelling and for investigating the presence of recording artefacts. This model offers relative simplicity and the advantage of the possibility of artefact-free recording. The purpose of this study was to characterize the artefact with impedance recording in unmyelinated peripheral nerve and address the following questions: (1) what are the characteristics of the artefact? (2) At what current density does the artefact become significant? (3) What are the implications for recording the fast neural impedance signal for EIT for recordings in the brain?

### 1.3. Experimental design

The experimental setup comprised an excised crab nerve, which was placed on a linear electrode array (figure 1). Electrodes numbered 1 and 2 were connected to the stimulator, which triggered the CAP propagation along the nerve. The current source was connected to the electrodes 5 and 6, and the voltage was measured between electrode 4 and one of the remaining electrodes. The electrode 3 is grounded in order to prevent stimulation artefact. The signal processing summation-subtraction method has been employed in order to separate CAP and impedance recordings which is explicitly described in (Oh *et al* 2011).

**Figure 2.**
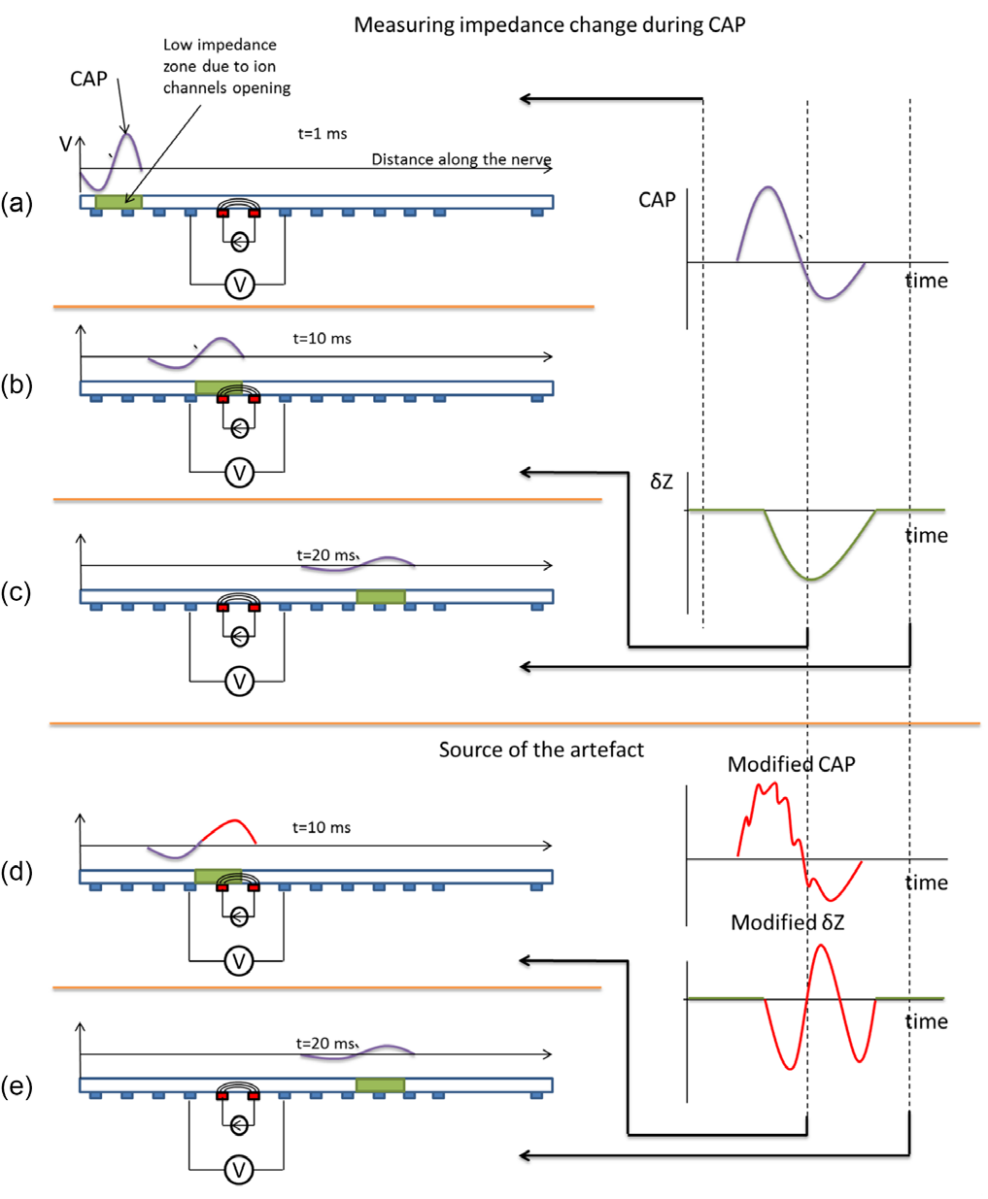
Illustration of artefactual changes in the crab nerve. The CAP propagates along the nerve together with the conductivity change perturbation caused by ion channels opening, and its amplitude gradually decreases towards the end of the nerve (*a*–*c*). If it is affected by the current when passing through the area where the current is injected (*d*, *e*), the raw measured voltage comprises the standing (background) potential modulated by the impedance change and is superimposed with the CAP, which is impossible to eliminate with averaging and subtraction, because the modified CAP contains the exact frequency and phase of the injected current (*e*). The resulting demodulated voltage will be contaminated by these changes, if measured with the electrodes located distally to the current injecting electrodes (*d*). The effect is negligible by the electrode 19 at end of the nerve where CAP is negligibly small and so cannot affect the measurements.

Voltage recording was with respect to electrode 4 and one other electrode distal to the impedance measuring current injection pair. The effect of CAP modification by applied current decreased as the distance between the reference electrode and current injecting electrodes increases, as the amplitude of CAP decreased with distance due to dispersion (figure 2). The artefact became negligible with recording at electrode 19 as the CAP was also negligible at that distance.

The artefact-free method was validated through the following controls: (1) all recordings at electrode 4 were compared to the recordings at electrode 3 in order to make sure that there was no significant difference in the impedance change and (2) all recordings at electrode 19 were compared to the closest to the injection site (electrodes 12–18) for the same reason. If any of the recordings were affected by the artefact, then controls (3–19 versus 4–19, and 4–19 versus 4–12(−18)) would have shown a significant difference. At the same time, the artefact-free recordings were compared to the simulations, which produce results with artefact-free impedance change, and a significant mismatch of the waveform, timing, or polarity of the impedance change between experimental and simulated results would reveal the presence of artefacts in experimental artefact-free recordings.

The presence of the impedance artefact was assessed by subtraction of measurement 4–19, from other recordings, such as 4–7, where the distal electrode recorded a significant CAP and so led to a significant artefact. The resulting change was then investigated in relation to the injected current frequency, amplitude, phase, and location. Three combinations of injecting electrodes were used (5–6, 6–7, and 7–8) in order to investigate consistency of the CAP artefact with respect to injecting position.

The values for current amplitude were set between the minimal SNR (5 *µ*A), and maximum possible current, above which the CAP was significantly altered and suppressed (20 *µ*A). The values for the phase were selected between 0 and 45° as a representative of the demodulation technique as in- and anti-phase current injections were both used for single measurements.

The underlying explanation for the artefact and its behaviour was also evaluated by comparison of the experimental findings with modelling of the genuine impedance change effect. Due to the physiological variability and uncertainty of the exact membrane capacitance values, two models were used: equivalent resistor network, and finite element model (FEM) of the nerve. Resistor network approximated changes at dc without modelling the frequency dependence, while FEM was used to approximate the relationship between the amplitude of the change and frequency. Modelling results were used as the bounds for the experimental impedance change.

## 2. Methods

### 2.1. Simulation methods

**Figure 3.**
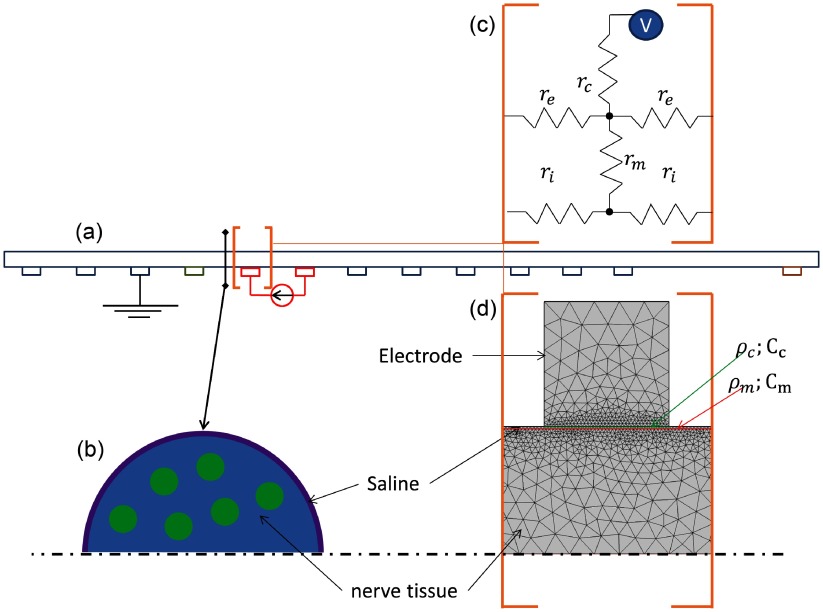
Resistor model and FEM used to simulate impedance change propagation in the nerve. For the resistor model, the nerve (*a*) was modelled as a long cable, discretized at each electrode (*c*) with intracellular resistance (*r*_i_), extracellular resistance (*r*_e_), membrane resistance (*r*_m_), and contact impedance (*r*_c_). In the FEM, the heterogeneous impedance of the nerve fibres was mapped (*b*) into the effective homogeneous impedance of the axisymmetric cylinder (*d*), with intra- and extracellular resistance (*ρ*_i_, *ρ*_e_), membrane impedance (*ρ*_m_, *C*_e_), contact impedance (*ρ*_c_, *C*_c_), and electrode resistivity (*ρ*_e_). During the activity, the perturbation with parameters (*ρ*_p_, *C*_p_, *r*_p_) moved with constant velocity 2 m s^−1^ along the nerve.

The membrane impedance change was modelled as a travelling perturbation with a length of 10 mm, which was determined on the basis of the experimental CAP space constant. The perturbation had constant resistivity across the length. It was 2–10 times smaller than the resistivity of unperturbed membrane (Liston *et al* 2012) (table 1). It was moving in a heterogeneous media with constant velocity of 2 m s^−1^, determined from the experimental results. The resting and perturbed membrane resistivity and shape did not change with time. Two models were investigated in order to predict expected impedance changes: a resistor model, and a FEM of the nerve. The resistor model was based on classical cable theory (Hodgkin and Huxley 1952) with variable membrane resistivity during the action potential conduction, and Kirchhoff’s laws were solved to predict voltage outcome on the electrodes. The FEM took into account contact impedance and heterogeneous nature of the resistivity, as well as capacitive effects (figure 3). Quasi-static time-harmonic current conduction problem was then solved for each time point:

\begin{eqnarray*}\begin{array}{*{35}{l}} \nabla \cdot \left(\sigma -\text{i}\omega \varepsilon \nabla u\right)=0 &amp; \quad \text{in}\text{}\Omega \\ {{{\int}^{}}_{{{\Gamma}_{l}}}}\sigma \frac{\partial u}{\partial \nu}\text{d}{{\Gamma}_{l}}={{I}_{l}} &amp; \quad l=1,\ldots ,M \\ u+\left({{\rho}_{l}}+\text{i}\omega {{C}_{l}}\right)\sigma \frac{\partial u}{\partial \nu}={{U}_{l}} &amp; \quad \text{on}\text{}{{\Gamma}_{l}},\quad l=1,\ldots ,M \\ \begin{array}{*{35}{l}} \nu \cdot \left({{J}_{1}}-{{J}_{2}}\right)=\left(\frac{1}{{{\rho}_{\text{m}}}}+\text{i}\omega {{C}_{\text{m}}}\right)\left(u-{{u}_{\text{ext}}}\right) \\ \sigma \frac{\partial u}{\partial \nu}=0 \\ \end{array} &amp; \begin{array}{*{35}{l}} ~~~~on~{{\Gamma}_{\text{m}}} \\ \text{}\text{}\text{}\text{}\text{}\text{ on}\text{}\Gamma \setminus \underset{l=1}{\overset{M}{\cup}} {{\Gamma}_{l}}{\cup}^{}{{\Gamma}_{\text{m}}}, \\ \end{array} \\ \end{array}\end{eqnarray*}

where *σ* is the conductivity in the domain, *ρ*_m_ is the membrane resistivity, *C*_m_ is the membrane surface capacitance, *u* is the electric potential in domain Ω, *ω* and *I* is the frequency and amplitude of applied current, ${{\left({{U}_{l}}\right)}_{l=1,\ldots ,M}}\in {{\mathbb{R}}^{M}}$ is the vector of the electric potentials on the electrodes abiding to the grounding condition $\underset{l=1}{\overset{m}{\sum}} {{U}_{l}}=0$, $\nu $ is the outward unit normal to surface Γ, *ε* is the electrical permittivity, *ρ*_*l*_ and *C*_*l*_ is the contact resistivity and surface capacitance on the electrode surfaces Γ_*l*_, and *J*_1_ – *J*_2_ is the current density across the membrane surface Γ_m_. For all models the exact procedure of impedance change extraction was applied (see 2.5). The parameters were taken from (Liston *et al* 2012) (table 1).

**Table 1.** Simulation parameters.

Resistor model parameters								
*r*_c_		*r*_m_		*r*_i_		*r*_e_		*r*_p_
200 Ω		10 000 Ω		300 Ω		500 Ω		1000 Ω
FEM parameters								
*ρ*_c_	*C*_c_	*ρ*_m_	*C*_m_	*σ*_in_	*σ*_out_	*σ*_el_	*ρ*_p_	*C*_p_
10^−6^ Ω m^2^	0 F m^−2^	0.8 Ω m^2^	0.01 F m^−2^	1.11 S m^−1^	5 S m^−1^	10^4^ S m^−1^	0.4 Ω m^2^	0.01 F m^−2^

### 2.2. Biological preparations

The walking leg nerve of the edible crab (*Cancer pagurus*) was dissected out and bathed in Crab Ringer solution at 4 °C. For each measurement, the nerve was blotted and placed on the electrode array for 1 min. The entire array was kept at a temperature of 4 °C by bathing in ice water. Further details are as in (Oh *et al* 2011).

### 2.3. Setup and hardware description

The 19-electrode array used for impedance measurements (figure 1) comprised two stimulation electrodes (1 and 2) for initiation of the CAP, a ground electrode (3) for removing stimulation artefacts, and measurement electrodes (4–19). A constant current was applied to 5–6, 6–7, or 7–8, and voltage was measured differentially with respect to the reference electrode 19 from 7–18. The measurement system comprised a 128 BioSemi multichannel data acquisition system with 16 kHz sampling frequency and 1 TOhm input impedance (BioSemi, Netherlands), and custom made isolated bipolar sine wave current source with maximal current of 20 *µ*A and output impedance of >100 k. The CAP was triggered by 0.1 mA electric current pulses with 0.2 ms duration produced with Neurolog NL800A stimulator (Digimeter, UK).

### 2.4. Experimental protocols

Data was acquired with continuous current injection and repeatedly stimulated responses, with 120 stimuli and 500 ms inter-stimulus time, which resulted in a total of 1 min for each individual recording. Consecutive stimulations were set to be in phase and in anti-phase with the injected current.

In each of 12 nerves, 2–3 individual recordings were made for each injected current frequency of 225, 625, and 1025 Hz, amplitudes of 5, 10, and 20 *µ*A, and phases of 0, 20, and 40° with respect to stimulation pulse, with current injected at electrodes 5–6 (272 recordings). In four nerves, current was also injected at 425 and 825 Hz (20 recordings). At 225 Hz in each of 12 nerves, two recordings were made where current was injected also between electrodes 6–7 or 7–8 (48 recordings). The total number of recordings was therefore 340.

In all recordings, voltages were acquired simultaneously between electrodes 3 : 18 with respect to 19, including current injecting electrodes. Filtering, demodulation, averaging, and measurement combination were performed digitally after data acquisition.

### 2.5. Signal processing

All impedance changes (*δZ*) were of the modulus computed from each voltage recording as follows (figures 2(*b*) and (*c*)): (1) the measurement was segmented into individual trials of 0.5 s around the stimulation trigger then (2) consecutive in-and anti-phase trials were (a) summed to yield the CAP and (b) subtracted to cancel the CAP and yield the impedance-modulated sine wave. (3) Each sine wave was band-pass filtered around the carrier (100 Hz band for 225 Hz, and 250 Hz band for the rest of the frequencies), then was demodulated using the modulus of Hilbert transform to yield the modulus of the complex impedance and (4) all impedance trials were averaged together. All individual measurements used in the analysis produced significant impedance changes (*P* < 0.001, *n* = 60 paired trials in each measurement, two-sided *t*-test). This method eliminated any additive voltage changes, even correlated ones, except those which arose from the current itself. All results are presented as mean ± 1 standard deviation (SD). Where it is not stated, the significance of each group of measurements test was tested using a two-tailed *t*-test.

### 2.6. Artefactual δZ extraction and characterization

The occurrence of genuine artefact-free impedance changes during the CAP was evaluated by ensuring that there were no significant differences between the following results: (1) recording with voltages from electrodes 4–19, 4–18 or 4–17, with current injection at 4–5 (on the basis that the CAP was fully dispersed at electrodes 17–19), (2) Recording with voltages from 4–19, 5–19 or 6–19, with current injection at 7–8. (3) Results from simulation with both the resistor and FEM model. The genuine impedance change was then subtracted from artefact-affected measurements. The resulting peak absolute artefactual impedance changes were then analysed with respect to measuring current amplitude, phase, and the injection site.

## 3. Results

### 3.1. Artefact-free recordings and controls

**Figure 4.**
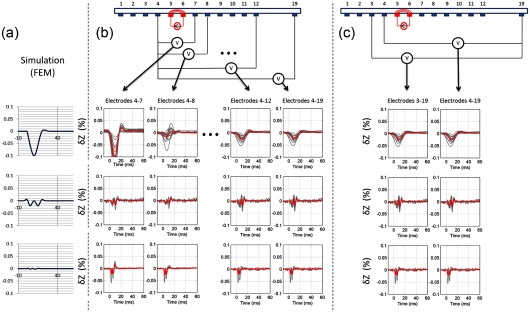
Summary of the recordings and controls taken at different frequencies. The artefact-free recordings (electrodes 4–19) were validated by comparing with simulations (*a*), recordings made with the last electrode being closer (*b*), and the first electrode further away (*c*) from the injection site. For the artefact-free method, there was no significant difference between consecutive pairs either way. The recordings, affected by the artefact can be clearly distinguished (electrodes 4–7 and 4–8, b). Grey traces represent each individual measurement, and the thick red trace represents the average impedance measurement across 30 recordings in 12 nerves.

The CAP diminished from 6 ± 2 mV (*n* = 216 in 12 nerves, *p* < 0.001) at electrode 3 to 0 ± 0.01 mV at 12 (*n* = 216 in 12 nerves, *p* > 0.05). The CAP was no greater than noise of 0.01 mV at electrodes 12–18. In artefact-free recordings (current at 4–5 and recording at 4–19), consistent impedance changes (*δZ*) of −0.045 ± 0.01% (*n* = 24 in 12 nerves, *p* < 0.001) occurred at 225 Hz. There were no significant differences to voltage measurement 3–19 and 4–19 (*n* = 24 in 12 nerves, *p* > 0.05) (figure 4(*c*)). There were no significant differences to voltage measurement 4–19 and 4–12 (*n* = 24 in 12 nerves, *p* > 0.05) (figure 4(*b*)).

The peak amplitudes of the modelled and experimental changes were (experimental {modelled FEM, modelled resistor network}) −0.045 ± 0.01% {−0.1%, −0.05%}, −0.02 ± 0.01% {−0.02%, −0.05%}, −0.02 ± 0.01% {−0.005%, −0.05%}, at 225 Hz, 625 Hz and 1025 Hz respectively (*n* = 24 in 12 nerves for experimental results). As expected, the resistor model cannot predict the amplitude-frequency decay correctly, but experimental results matched at lower frequencies (225 Hz). The FEM model predicts sharper decay, which was probably due to the physiological variations of the actual membrane capacitance. However, the experimental results were well in range between the predicted values of two models, and the waveform timing and shapes were similar between experimental and simulated results for all frequencies (figure 4(*a*)).

### 3.2. Artefact characterization

The occurrence of artefactual changes was evaluated in recordings with measuring current injected through 5 and 6, and voltage recording with electrodes 7–19 with respect to 4. *δZ* recordings appeared to be artefact-free with voltage recording from electrodes 12, 9 or 7 distally for recording frequencies of 225 Hz, 625 Hz and 1025 Hz respectively (figure 4(*b*)) as there was no significant difference between *δZ* for recordings with a distal second voltage recording electrode, taken individually for each applied current frequency (*P* > 0.05, *n* = 24 in 12 nerves, one-way ANOVA). With a second voltage recording electrode placed proximally to these, *δZ* differed from the artefact-free recordings by −0.6–0.5% or −0.005–0.004%, for 225 Hz (pairs 4–7 to 4–11) and 625 Hz (pairs 4–7 and 4–8) respectively (figure 4(*b*)).

**Figure 5.**
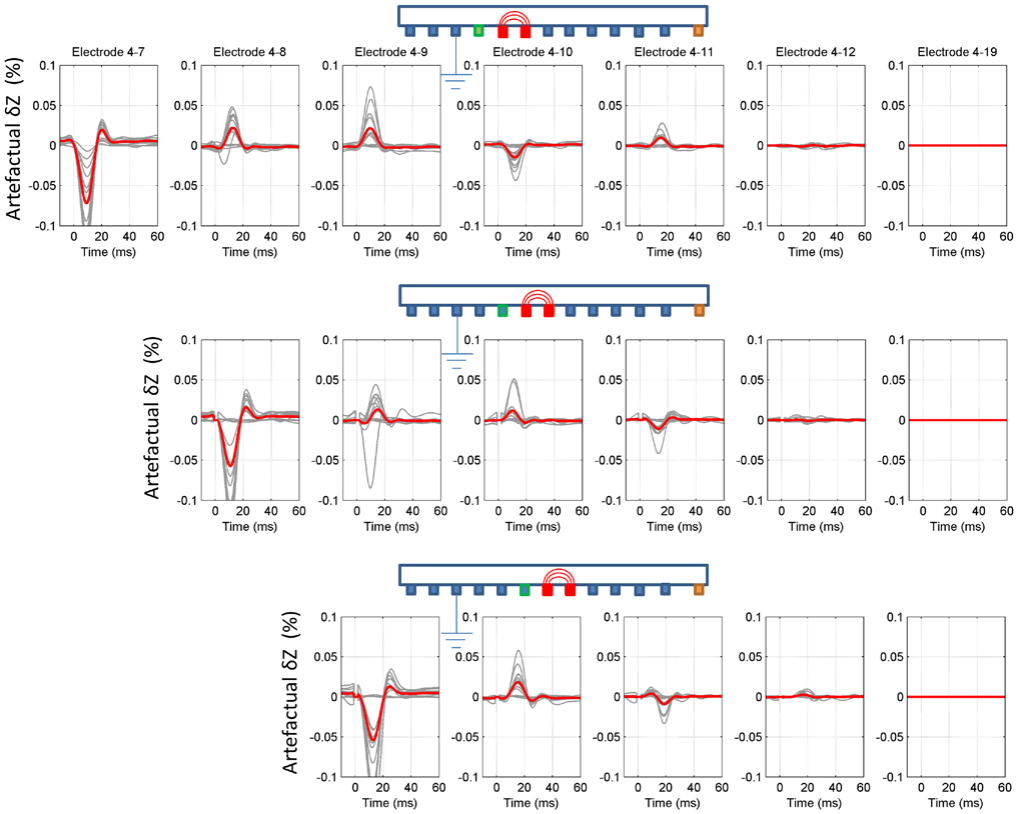
Extracted artefactual changes at 225 Hz (the largest artefact) at different current injection locations. Top row shows measurements with current being injected between electrodes 5–6, middle row—electrodes 6–7, and bottom row—electrodes 7–8. Grey traces represent individual measurements, and the red trace is the average across 24 measurements in 12 nerves.

**Figure 6.**
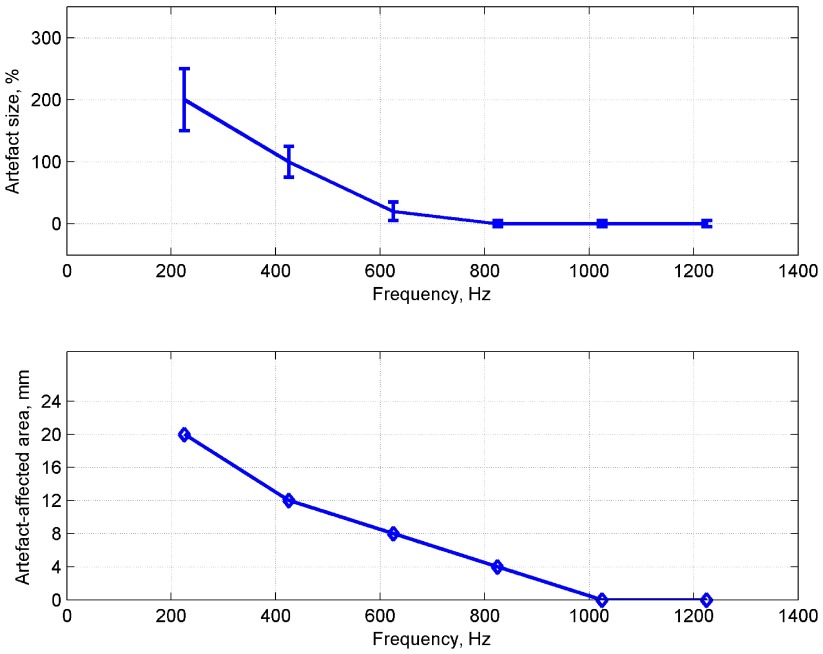
Artefactual changes with respect to frequency. The peak artefact in % with respect to the genuine impedance size (top, mean and standard deviation, *N* = 10 in four nerves), and artefact-affected area (bottom), approximated as the distance to the electrode where artefact becomes non-significant across *N* = 10.

The net artefactual changes were evaluated by subtraction of the artefact-free *δZ*, with voltage recording at 4–19, from that with a more proximal second voltage electrode. These were reproducible and consistent with current injected through pairs 5–6, 6–7 and 7–8 (*p* < 0.001, *n* = 24 in 12 nerves for each injection pair, two-way ANOVA) (figure 5). The peak *δZ* and area of the artefactual changes decreased with respect to frequency; it was negligible above 1 kHz (*P* > 0.05, *n* = 24 in 12 nerves) (figure 6).

**Figure 7.**
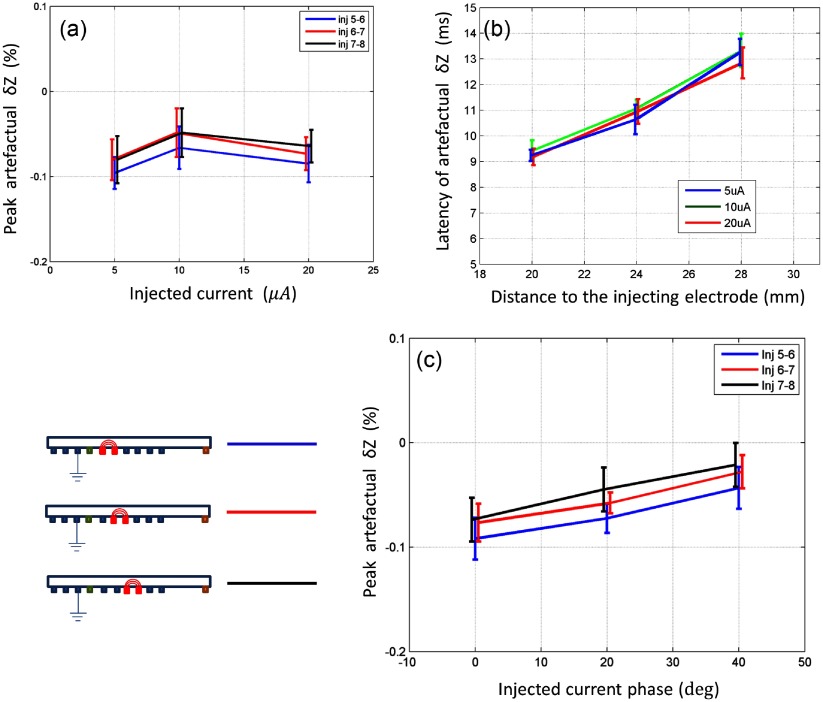
Net artefactual change analysis performed at 225 Hz. (*a*) Peak amplitude of the artefactual changes with respect to the injected current amplitude, mean and standard deviation (*n* = 10 in four nerves for each value, colours represent different injection electrodes), (*b*) latency of the peak of artefactual change with respect to the distance between stimulating and current injecting electrode (*n* = 8 in four nerves for each value, colour lines represent different current amplitudes), and (*c*) Peak amplitude of the artefactual changes with respect to the current injection phase, mean and standard deviation (*n* = 10 in four nerves for each value, colours represent different injection electrodes).

The proportional amplitude (*δZ* in %) of the artefact did not increase significantly with the amplitude of the injected current (*P* > 0.05, *n* = 10 in four nerves for each value) (figure 7(*a*)), and the latency of onset was proportional to the distance between the stimulating and current injecting electrodes (*n* = 8 in four nerves) (figure 7(*b*)). However, the amplitude of the artefact significantly correlated with the phase (*P* < 0.01, *n* = 10 in four nerves) and was decreasing from −0.09 ± 0.024 to −0.03 ± 0.023% with phase of 0 to 45° (figure 7(*c*)).

## 4. Discussion

### 4.1. Summary of results

Significant artefactual changes occurred in recording impedance changes during the CAP if voltages were recorded either side of the current measuring electrodes, as the distal voltage electrode recorded a CAP which was altered by the injecting current. The amplitude of the artefact reached approximately 300% and 50% of the genuine membrane impedance change at 225 Hz and 600 Hz respectively. Both the artefactual *δZ*, and the CAP recovered after bandpass filtering for impedance demodulation, diminished with the current measuring frequency, and were not significant above 1 kHz. The artefactual proportional *δZ* changes (expressed as % change from baseline) were independent of the measuring current amplitude in the range 5–20 *µ*A and their latency varied with the position of the impedance measuring current injection electrodes. In this respect, they behaved like genuine impedance changes. However, the magnitude of artefactual *δZ* decreased with the phase of the impedance current.

### 4.2. Current level at which the artefact occurred

In order to generate a satisfactory SNR, the lowest current used in this study was 5 *µ*A. At this level, the artefact was already present. The use of these currents, however, did not significantly affect the CAP. It still clearly propagated along the nerve, and its basic parameters were not affected.

It was unexpected that the size of the proportional artefactual changes did not change with the applied measuring current. This suggests that the change in the potential component of the CAP, induced by the current, was proportional to the amplitude of the current. An analysis of the ionic components of such a change is outside the scope of this paper, but data, obtained with patch-clamp technique indicates that most channel properties are non-linearly related to trans-membrane potential (Methfessel *et al* 1986). This linear effect is presumably caused by a linear effect on ion channels leading to the CAP, which makes the artefactual changes behave like the genuine impedance change.

### 4.3. Implications for EIT of brain function

Although the artefact was linearly related to the amplitude of applied current in the range used in this study, it did vary with the phase of the applied current. This suggests that there was a specific temporal region of the CAP which was affected by the current, which passes through the signal processing stage. The practicality of this in application to EIT of the brain is limited: depending on the location of the activity, and its onset after stimulation, the relationship between the activity and the current will be different as the activity propagates in a comparatively large volume, commonly occurring in multiple, areas simultaneously, and spans different points in time. Therefore precise phase control, as would be suggested by results (figure 7(*b*)) is impractical. However, if the phase is randomized, then given the fact that there is direct relationship, the artefact should at least be reduced, or completely average out, because at every affected spatial point it will behave as a temporally random noise.

The size of the artefactual change is negligible at higher frequencies. This suggests that the neural activity is not sensitive to those frequencies, which can be indirectly confirmed by the spectral analysis of CAP. In previously reported studies, applied frequencies of up to 225 Hz were used, and the main controls were to determine if the artefactual *δZ* was linear and the CAP or evoked potentials did not alter with applied current. These findings suggest a different approach in further studies in EIT of fast neural activity of brain function:

(a) The frequency of the current should be greater than 1 kHz. Modelling at least in crab nerve (Liston *et al* 2012) has suggested that *δZ* is about 100 × smaller at this frequency than at dc but the component of the evoked potentials or the EEG for brain recordings is also much smaller, so this may still lead to a significant improvement in SNRs. It may also be that the decrease in *δZ* with frequency falls less with recording in the brain where cell geometry differs from that in nerve.
(b) For recordings with applied currents below 1 kHz, the relation of the impedance change with respect to applied current should still be investigated. If non-linear, this points to the presence of artefactual *δZ*. However, linearity of *δZ* with respect to current cannot be taken as strong evidence for the absence of the artefact.
(c) Comparison with biophysical modelling and controls with checking of effect of applied current on the evoked potentials should still be employed.
(d) The phase of the injected current should be randomized with respect to the stimulation during impedance recordings. Although it is hard to predict without knowing the exact relationship between the artefact and the phase, this should minimize the effect of the artefactual change with averaging as any artefact will be reduced or cancel. In addition, control recordings against varying phase should be undertaken.

### 4.4. Study limitations and future work

The relationship between the artefact and the phase of the injected current with respect to the stimulus is a surprising and very interesting finding, however to fully analyse this effect, more experiments are required with phases spanning 0–180°, which was not possible due the limitation of the current hardware. More experiments with enhanced hardware are planned in order to fully investigate this effect. At the same time, the *in vivo* experiments are also planned in order to access this effect in the brain tissue as the response is expected to be physiologically different.

## References

[pmea511146bib0001] Cole K S, Curtis H J (1939). Electric impedance of the squid giant axon during activity. J. Gen. Physiol..

[pmea511146bib0002] Edwards D, Cortes M, Datta A, Minhas P, Wassermann E M, Bikson M (2013). Physiological and modeling evidence for focal transcranial electrical brain stimulation in humans: a basis for high-definition tDCS. Neuroimage.

[pmea511146bib0003] Freygang W H, Landau W M (1955). Some relations between resistivity and electrical activity in the cerebral cortex of the cat. J. Cell. Comp. Physiol..

[pmea511146bib0004] Galambos R, Velluti R (1968). Evoked resistance shifts in unanesthetized cats. Exp. Neurol..

[pmea511146bib0005] Gilad O, Ghosh A, Oh D, Holder D S (2009). A method for recording resistance changes non-invasively during neuronal depolarization with a view to imaging brain activity with electrical impedance tomography. J. Neurosci. Methods.

[pmea511146bib0006] Gilad O, Holder D (2009). Impedance changes recorded with scalp electrodes during visual evoked responses: implications for electrical impedance tomography of fast neural activity. Neuroimage.

[pmea511146bib0007] Gilad O, Horesh L, Holder D S (2007). Design of electrodes and current limits for low frequency electrical impedance tomography of the brain. Med. Biol. Eng. Comput..

[pmea511146bib0008] Hodgkin A L, Huxley A F (1952). A quantitative description of membrane current and its application to conduction and excitation in nerve. J. Physiol..

[pmea511146bib0009] Holder D S (1987). Feasibility of developing a method of imaging neuronal activity in the human brain: a theoretical review. Med. Biol. Eng. Comput..

[pmea511146bib0010] Holder D S (1992). Impedance changes during the compound nerve action potential: implications for impedance imaging of neuronal depolarisation in the brain. Med. Biol. Eng. Comput..

[pmea511146bib0011] Liebetanz D, Koch R, Mayenfels S, König F, Paulus W, Nitsche M A (2009). Safety limits of cathodal transcranial direct current stimulation in rats. Clin. Neurophysiol..

[pmea511146bib0012] Liston A, Bayford R, Holder D (2012). A cable theory based biophysical model of resistance change in crab peripheral nerve and human cerebral cortex during neuronal depolarisation: implications for electrical impedance tomography of fast neural activity in the brain. Med. Biol. Eng. Comput..

[pmea511146bib0013] Merton P A, Morton H B (1980). Stimulation of the cerebral cortex in the intact human subject. Nature.

[pmea511146bib0014] Methfessel C, Witzemann V, Takahashi T, Mishina M, Numa S, Sakmann B (1986). Patch clamp measurements onXenopus laevis oocytes: currents through endogenous channels and implanted acetylcholine receptor and sodium channels. Pflgers Arch. Eur. J. Physiol..

[pmea511146bib0015] Oh T, Gilad O, Ghosh A, Schuettler M, Holder D S (2011). A novel method for recording neuronal depolarization with recording at 125–825 Hz: implications for imaging fast neural activity in the brain with electrical impedance tomography. Med. Biol. Eng. Comput..

[pmea511146bib0016] Olney R K, Budingen H J, Miller R G (1987). The effect of temporal dispersion on compound action potential area in human peripheral nerve. Muscle Nerve.

[pmea511146bib0017] Reato D, Rahman A, Bikson M, Parra L C (2013). Effects of weak transcranial alternating current stimulation on brain activity-a review of known mechanisms from animal studies. Front. Hum. Neurosci..

[pmea511146bib0018] Smith T G, Wuerker R B, Frank K (1967). Membrane impedance changes during synaptic transmission in cat spinal motoneurons. J. Neurophysiol..

[pmea511146bib0019] Tsukahara N, Fuller D R (1969). Conductance changes during pyramidally induced postsynaptic potentials in red nucleus neurons. J. Neurophysiol..

